# POLICY SERIES: ELDER ABUSE AND THE OPIOID EPIDEMIC IN RURAL AMERICA

**DOI:** 10.1093/geroni/igz038.2801

**Published:** 2019-11-08

**Authors:** Brian W Lindberg, Robert Blancato

**Affiliations:** 1 The Gerontological Society of America, Washington, District of Columbia, United States; 2 Matz, Blancato & Associates, Washington, District of Columbia, United States

## Abstract

Misuse of opioids is a national crisis affecting the social and economic welfare of communities throughout the U.S. and is particularly rampant in rural America. Older adults are far too frequently excluded from consideration of those who are affected by the opioid epidemic. While rural older adults may not suffer the highest per capita rate of opioid overdose deaths, they are deeply affected by the problem. In their youth and middle adulthood, many older adults used their bodies for labor. At older ages, they experience multiple chronic conditions and high rates of chronic pain for which opioids and related prescription and non-prescription drugs are often the treatment of choice. Also and far too frequently, older people become easy targets for abuse by persons needing resources to feed their addiction. This symposium focuses on elder abuse associated with opioid and related substance misuse. Zanjani’s presentation provides the context of rurality and drugs and alcohol as a precursor to elder abuse. The second paper by Teaster and colleagues examines trends in APS cases of elder abuse in which the perpetrator is a substance user and identifies perpetrator and victim characteristics predictive of different types of substantiated abuse. Using APS case notes, Roberto and colleagues characterize cases of elder abuse in rural Kentucky in which the perpetrator used opioids and related substances. Robert Blancato and Brian Lindberg will discuss presenters’ collective findings by weaving together concepts of rurality, addiction, and elder abuse and recommending strategies for prevention, intervention, and policy.

